# Vertebral Discitis in a Young Patient With No Comorbidities Caused by Salmonella enterica Serovar Agbeni Infection: A Case Report

**DOI:** 10.7759/cureus.29773

**Published:** 2022-09-30

**Authors:** Daniela Pi Noa, Rodrigo Podesta, Joshua Costin, Soling Li

**Affiliations:** 1 Medicine, Nova Southeastern University, Dr. Kiran C. Patel College of Osteopathic Medicine, Fort Lauderdale, USA; 2 Medical Education, Nova Southeastern University, Dr. Kiran C. Patel College of Allopathic Medicine, Fort Lauderdale, USA; 3 Internal Medicine, Nova Southeastern University, Dr. Kiran C. Patel College of Osteopathic Medicine, Fort Lauderdale, USA

**Keywords:** osteomyelitis, osteodiscitis, salmonella enterica discitis, vertebral discitis, salmonella enterica

## Abstract

Vertebral discitis is an infection of the vertebrae most commonly caused by *Staphylococcus aureus *(*S. aureus*) and usually presents in patients with preexisting medical conditions such as diabetes mellitus. This disease process involves the invasion of bacteria into the vertebral disc through one of three basic routes: hematogenous spread from a distant site, direct trauma due to iatrogenic causes, or due to a contiguous spread from adjacent soft tissue infection. Here, we present a 24-year-old Asian male with no past medical history or history of trauma who presented with nonspecific symptoms of fever and nasopharyngitis. The patient subsequently developed persistent thoracic back pain that failed multiple lines of treatment. Magnetic resonance imaging (MRI) of the spine showed vertebral discitis, and cultures confirmed *Salmonella* being the etiologic agent for his symptoms. *Salmonella* discitis is extremely rare, with only about 0.45% of these cases being reported in the literature. Even more uncommon is the isolation of *Salmonella enterica* serovar Agbeni in a young patient without comorbidities. This case report highlights the importance of including *Salmonella* as a possible causative agent in patients who present with signs and symptoms of vertebral discitis.

## Introduction

Vertebral discitis refers to the infection of vertebral discs, and it is prominently found in the adult population over the age of 50 [1]. There are three sources of infection: hematogenous spread, direct inoculation, or contiguous spread from an adjacent source, with the hematogenous spread being the most common source [2]. The most common pathogen causing vertebral discitis is* Staphylococcus aureus* (*S. aureus*) [3]. Nontyphoid salmonella typically presents as gastroenteritis and constitutes a very rare source of vertebral discitis. When found to be the causative agent, it is generally isolated as the cause of discitis after the inoculation of a traumatic wound.

Only 1% of nontyphoidal salmonella infections are isolated in the urine and are most likely seen in patients over 60 years of age or immunocompromised patients. Moreover, *Salmonella enterica* serovar Agbeni is a rare type of salmonella species with only eight cases reported in Canada between 2000 and 2010 [4]; the CDC reported 76 cases in the United States in 2017, and 56 cases were reported in Europe between 2018 and 2019 [5]. It was first reported as a cause of osteomyelitis in 2017 in a 58-year-old male after an outbreak [6].

Patients with vertebral discitis usually present with spinal pain that is initially localized to the infected spinal disc and worsens over time. Half the patients experience fever [7]. Laboratory findings will show elevated erythrocyte sedimentation rate (ESR) and C-reactive protein (CRP) in 80% of patients [8]. Magnetic resonance imaging (MRI) is the radiologic study of choice for diagnosing vertebral discitis, and diagnosis can be confirmed by positive cultures [9].

The management of this condition consists of the eradication of the causative agent, and thus, antimicrobial therapy plays a crucial role in the treatment. Early intervention prevents the further spread and detrimental course of the disease in patients with vertebral discitis. Early empiric treatment is usually started after cultures have been collected to prevent neurological degeneration. The antimicrobial agent used to treat this condition is highly specific to the results of biopsy and cultures, as well as antimicrobial sensitivity [7]. First-line treatment for the most common etiologic agent,* Staphylococcus aureus*, consists of intravenous (IV) vancomycin and a third- or fourth-generation cephalosporin. On the other hand, the preferred treatment for *Salmonella* discitis consists of systemic fluroquinolones with ampicillin and third-generation cephalosporins [7]. Patients remain on systemic antibiotics for at least six weeks, and inflammatory markers should be monitored on a weekly basis, as normalization of ESR and CRP has been found to be highly correlated to successful treatment [8]. Analgesics also play a role in the treatment and are often used for pain management [7].

## Case presentation

We present a 24-year-old Asian male with a high-grade fever of 101°F-102°F being evaluated in the outpatient setting, initially treated for nonspecific fever and nasopharyngitis with oseltamivir phosphate and methylprednisolone. The patient returned to the clinic one week later with a persisting fever of 101°F-102°F and new-onset back pain. He was placed on acetaminophen codeine for back pain at this time. Two weeks later, the patient complained of worsening back pain and new onset of intermittent urinary frequency and urgency. Office urine dipstick was positive for leukocytes and nitrites, and trimethoprim-sulfamethoxazole 160 mg was prescribed for seven days to treat the urinary tract infection. Diclofenac 50 mg twice a day for back pain was added to the treatment with no resolution of symptoms. In the following month, the patient had one urgent care visit with negative findings on a thoracic-lumbar X-ray. In the following week, the patient went to the emergency department with complaints of back pain and was discharged home with physical therapy follow-up. The patient returned to the emergency department three weeks later with the same symptoms. X-rays of the thoracic and lumbar spine were done at each emergency department visit, which were negative eight weeks post initial presentation of symptoms. At the twelfth week from the onset of his original symptoms, the patient failed to improve with physical therapy and returned to the office where he was prescribed oxycodone and acetaminophen, baclofen, diclofenac sodium, and topical Voltaren gel for symptomatic treatment of back pain. He was also referred to neurosurgery for further evaluation and the management of his ongoing back pain. MRI of the lumbar spine was done in the outpatient setting, which showed osteodiscitis/osteomyelitis of T11-T12. The neurosurgeon contacted the patient and advised him to go to the emergency room for immediate treatment.

In the emergency department, the patient was stable. Upon arrival, his vitals showed 100% oxygen saturation on room air, blood pressure of 134/78 mmHg, temperature of 98.3°F, pulse rate of 78 beats per minute, and a respiratory rate of 18 breaths per minute. The review of systems was significant for back pain. The patient denied saddle anesthesia, incontinence, numbness, or weakness.

A focused physical exam of the back showed positive thoracic paraspinal tenderness and lumbar paraspinal tenderness. There were full range of motion, no signs of trauma, and no midline vertebral tenderness; straight leg raise test was negative, and there was no costovertebral angle tenderness. Laboratory findings are shown in Table 1 and were significant for decreased hematocrit, elevated lipase, and mildly elevated erythrocyte sedimentation rate.

**Table 1:** Laboratory values of the patient after admission to the emergency department. WBC: white blood cell; Hb: hemoglobin; Hct: hematocrit; BUN: blood urea nitrogen; Cr: creatinine; AST: aspartate aminotransferase; ALT: alanine transaminase; ESR: erythrocyte sedimentation rate.

	Patient’s values	Reference range
WBC (×10^9^/L)	8.0	5-10
Hb (g/dL)	13.7	13.5-17.5
Hct (%)	38.5	41-53
BUN (mg/dL)	18	7-18
Cr (mg/dL)	1.06	0.6-1.2
AST (U/L)	35	12-38
ALT (U/L)	15	10-40
Lipase (U/L)	211	10-140
ESR (mm/hour)	17	0-15

A chest X-ray showed no radiographic evidence of acute cardiopulmonary disease. An electrocardiogram (ECG) displayed a normal sinus rhythm and normal rate, with no acute ischemic changes. The patient was diagnosed with back pain and discitis of the thoracic region. He was started on intravenous piperacillin/tazobactam 3.375 g and vancomycin 1 mg empiric treatment based on the most likely organism (*S. aureus*) and admitted to the hospital for further evaluation and treatment.

Following admission, the patient was evaluated by an infectious disease specialist, and a lumbar puncture was done. Cerebrospinal fluid cultures were obtained to isolate the causative agent, and computed tomography (CT)-guided biopsy of spinal level T11-T12 was performed concurrently. The pathology report described fragments of fibrous tissue with acute and lymphoplasmacytic inflammation but was negative for malignancy and negative for fungal organisms. Cultures were positive for *Salmonella enterica* serovar Agbeni. Antibiotic therapy was adjusted based on the blood culture results. Piperacillin/tazobactam was stopped, vancomycin was continued, and the patient was started on IV ceftriaxone for one week in the inpatient setting, and a peripherally inserted central catheter (PICC) line was placed for outpatient antibiotics administration at home.

The patient followed up at the office one week after completing a seven-day course of ceftriaxone and being discharged with a diagnosis of vertebral discitis secondary to *Salmonella enterica* infection. All hospital records were reviewed, and the diagnosis was confirmed with the inpatient team. The patient presented to the office with a PICC line in place and receiving vancomycin 1,000 mg intravenous twice a day. During four weeks of intravenous antibiotic treatment, his back pain improved gradually.

## Discussion

Vertebral osteomyelitis is the inflammation and possibly infection of the bone in the vertebral column. On the other hand, vertebral discitis is considered to be the inflammation and possibly infection of the vertebral discs. Both of these disease processes can coexist or occur independently and are referred to as pyogenic spondylitis when presenting together [2]. Since the diagnosis and management of these two processes are similar, they are used interchangeably.

Vertebral discitis/osteomyelitis usually manifests in patients greater than 50 years of age, with incidence increasing progressively with age [10]. The vertebral discitis reported by the authors is rare due to the patient’s young age and the lack of risk factors for hematogenous spread. The patient presented with persistent fever and mid-to-lower back pain coexisting with an infection of the genitourinary system. This presentation made it difficult to discern when the possible vertebral infection began.

The pathogenesis surrounding vertebral discitis and vertebral osteomyelitis usually involves the hematogenous spread of the bacterium from a distant site to the new infection site, direct inoculation from trauma, or contiguous spread from adjacent soft tissue infection [3]. In this case, it is difficult to pinpoint the exact mechanism that led to the infection in the vertebral column since the common methods of pathogenesis seem unlikely in this case. Looking back at the history of illness from this patient in the outpatient setting, no trauma or contiguous spread from a soft skin lesion was present, leaving the hematogenous spread of bacterium as a source of infection as the main plausible causation. Similarly, the patient’s back pain began before his urinary symptoms, and the urine cultures during his hospital stay were negative, making genitourinary spread unlikely in this case.

During the hospital stay, it was discovered that the patient’s infection was caused by *Salmonella*. The *Salmonella* species are a rare cause of vertebral osteomyelitis, accounting for only 0.5% of all cases [4]. It is known that 54% of *Salmonella* osteomyelitis had predisposing conditions for infection, such as a medical history of sickle cell disease or long-standing diabetes mellitus, while the remaining 46% did not have any predisposition [11]. Our patient was not found to have any of the common risk factors for the development of *Salmonella* vertebral osteomyelitis, which include degenerative spinal disease, prior spinal surgery, and diabetes mellitus [11].

## Conclusions

In summary, this case outlines the presentation of osteodiscitis/osteomyelitis in an outlier patient population. It further portrays an extremely rare serovar (Agbeni) of an already rare causative agent (*Salmonella*) as the culprit of the patient's signs and symptoms. Moreover, it also highlights the importance of the timeline of this patient’s disease course of persistent high-grade fever followed by worsening back pain in the absence of injury or trauma as a possible indication of vertebral discitis/osteomyelitis. A high index of suspicion is warranted for early detection and further evaluation of this condition. This presentation should prompt further evaluation of spinal symptoms by means of magnetic resonance imaging (MRI) to rule out osteodiscitis/osteomyelitis. Furthermore, close surveillance of the patient’s progress is needed to prevent further complications that might lead to spinal surgery.
